# Specific ion channels contribute to key elements of pathology during secondary degeneration following neurotrauma

**DOI:** 10.1186/s12868-017-0380-1

**Published:** 2017-08-14

**Authors:** Ryan L. O’Hare Doig, Wissam Chiha, Marcus K. Giacci, Nathanael J. Yates, Carole A. Bartlett, Nicole M. Smith, Stuart I. Hodgetts, Alan R. Harvey, Melinda Fitzgerald

**Affiliations:** 1 Experimental and Regenerative Neurosciences, School of Biological Sciences, The University of Western Australia, Crawley, WA 6009 Australia; 2 Experimental and Regenerative Neurosciences, School of Human Sciences, The University of Western Australia, Crawley, WA 6009 Australia; 3 Experimental and Regenerative Neurosciences, School of Chemistry and Biochemistry, The University of Western Australia, Crawley, WA 6009 Australia; 4 Curtin Health Innovation Research Institute, Curtin University, Perth, WA Australia; 5 Perron Institute for Neurological and Translational Science, Verdun St, Nedlands, WA 6009 Australia

**Keywords:** Secondary degeneration, Neurotrauma, Traumatic injury, Ion channel inhibitor, Axonal degeneration, Node of Ranvier, Lipid peroxidation, Oligodendrocyte precursor cells, Oxidative stress

## Abstract

**Background:**

Following partial injury to the central nervous system, cells beyond the initial injury site undergo secondary degeneration, exacerbating loss of neurons, compact myelin and function. Changes in Ca^2+^ flux are associated with metabolic and structural changes, but it is not yet clear how flux through specific ion channels contributes to the various pathologies. Here, partial optic nerve transection in adult female rats was used to model secondary degeneration. Treatment with combinations of three ion channel inhibitors was used as a tool to investigate which elements of oxidative and structural damage related to long term functional outcomes. The inhibitors employed were the voltage gated Ca^2+^ channel inhibitor Lomerizine (Lom), the Ca^2+^ permeable AMPA receptor inhibitor YM872 and the P2X_7_ receptor inhibitor oxATP.

**Results:**

Following partial optic nerve transection, hyper-phosphorylation of Tau and acetylated tubulin immunoreactivity were increased, and Nogo-A immunoreactivity was decreased, indicating that axonal changes occurred acutely. All combinations of ion channel inhibitors reduced hyper-phosphorylation of Tau and increased Nogo-A immunoreactivity at day 3 after injury. However, only Lom/oxATP or all three inhibitors in combination significantly reduced acetylated tubulin immunoreactivity. Most combinations of ion channel inhibitors were effective in restoring the lengths of the paranode and the paranodal gap, indicative of the length of the node of Ranvier, following injury. However, only all three inhibitors in combination restored to normal Ankyrin G length at the node of Ranvier. Similarly, HNE immunoreactivity and loss of oligodendrocyte precursor cells were only limited by treatment with all three ion channel inhibitors in combination.

**Conclusions:**

Data indicate that inhibiting any of a range of ion channels preserves certain elements of axon and node structure and limits some oxidative damage following injury, whereas ionic flux through all three channels must be inhibited to prevent lipid peroxidation and preserve Ankyrin G distribution and OPCs.

## Background

Following trauma to the central nervous system (CNS), cells beyond the initial injury site succumb to degenerative events in a series of sequelae referred to as secondary degeneration. Secondary degeneration of white matter adjacent to a primary injury and vulnerable to damage can be modelled by partial transection of the optic nerve, where axons in the dorsal optic nerve are axotomised by the primary injury and ventral optic nerve is initially intact but vulnerable to secondary degeneration [1, 2]. Tracing of optic nerve axons using fluorescent dyes applied to ventral retina illustrates that ventral axons remain intact in the early stages following the partial transection [2]. Changes in Ca^2+^ flux occur following neurotrauma [3], and in optic nerve vulnerable to secondary degeneration following partial transection [4, 5]. Altered Ca^2+^ flux is associated with increased reactive oxygen species (ROS), enhanced anti-oxidant activity, and oxidative damage to DNA, protein and lipid in the first minutes and days following injury [6–8]. Cytosolic Ca^2+^ concentrations increase via influx from extracellular pools, and also via Ca^2+^-mediated release from intracellular stores [9, 10]. Ca^2+^ enters cells of the CNS through a range of channels and receptors, including but not limited to: voltage-gated Ca^2+^ channels (VGCCs) [11]; purinergic P2X_7_ receptors (P2X_7_Rs) [12]; and Ca^2+^ permeable ionotropic α-amino-3-hydroxy-5-methyl-4-isoxazolepropionic acid receptors (AMPARs) [13]. Intracellular levels of Ca^2+^ are typically low in neurons and glia, however excessive depolarization of neurons via glutamate excitotoxicity [14, 15] promotes intracellular Ca^2+^ influx through VGCCs [16]. Increasing extracellular ATP concentrations are also a typical consequence of injury [17], leading to increased Ca^2+^ flux through P2X_7_Rs [18].

Ca^2+^ influx following axonal injury activates deleterious cascades which induce the breakdown of cytoskeletal proteins and disruption of axonal transport, often leading to degeneration and neuronal death. Tau is a cytoskeletal phosphoprotein and although dynamic, site-specific phosphorylation of Tau is essential for its proper functioning, inappropriate and or hyper-phosphorylation at tyrosine 205 (Tau [pT^205^]), serine 262 (Tau [pS^262^]) and serine 396 (Tau [pS^396^]) [19, 20] renders it toxic and results in impaired microtubule assembly, disruption of anterograde and retrograde axonal transport and calpain mediated cell death [20–23]. The microtubule protein tubulin is acetylated in an activity-dependent manner via Ca^2+^ influx [24]. Reductions in acetylated tubulin are associated with Alzheimer’s disease and Parkinson’s disease [25, 26], and it is argued that tubulin acetylation may be a consequence of, rather than contributor to, microtubule stability. Limiting Ca^2+^ influx is neuroprotective via maintenance of the integrity of cytoskeletal proteins, however it is not known which cytoskeletal elements are essential for maintenance of axonal integrity, myelin structure and long term function following injury [27], and the timing of disruptions to axonal proteins during secondary degeneration is not yet clear.

Following injury, distribution of node of Ranvier and paranode proteins is also disrupted, contributing to functional impairment [28]. Caspr localization changes with spinal cord injury, with aberrant overlapping of Caspr, Kv1.1 and Kv1.2 in the paranodal regions [29]. Paranodal unfurling and lengthening of the paranode and paranodal gap are observed at 1 and 3 days as well as chronic time points in white matter vulnerable to secondary degeneration [30]. Myelin disruption is linked to its vulnerability to secondary events such as energy depletion, excitotoxicity [31], over-production of ROS and lipid peroxidation [32], which are elevated in optic nerve vulnerable to secondary degeneration [6]. Myelinating oligodendrocytes and oligodendrocyte precursor cells (OPCs) are particularly sensitive to excitotoxic insult and oxidative stress, with greater sensitivity of progenitor cells [33]. Early OPC proliferative responses and OPC depletion from 7 days are features of secondary degeneration [34], associated with myelin decompaction detected by electron microscopy from 3 months [35, 36]. However, mature oligodendrocyte numbers are maintained at relatively constant levels throughout [34]. The oligodendrocyte membrane protein NogoA is an inhibitor of axonal regeneration, located predominantly in the innermost and outer myelin membranes [37]. Studies demonstrate variable changes in the expression of NogoA following injury, with increases and decreases depending upon injury model and timing [38–40]. NogoA dynamics in secondary degeneration are unknown, yet the dynamic variation in NogoA expression could potentially explain different regenerative phenotypes and lack of a consistent therapeutic effect of NogoA inhibition [41].

Given the consequences of excessive Ca^2+^ during secondary degeneration, limiting Ca^2+^ entry into cells is hypothesized to reduce oxidative stress, and disruptions to axonal and myelin structure. The administration of single Ca^2+^ channel inhibitors have been used as a therapeutic strategy for CNS injury in vivo, reviewed in [42]. Whilst promising results have been demonstrated in pre-clinical studies, clinical trials have been disappointing [43, 44]. Given the multiple routes of entry of Ca^2+^ in the CNS, it is increasingly understood that a combinatorial approach to therapy, involving inhibition of multiple channels through which Ca^2+^ can pass, is likely to be required. We have previously assessed the efficacy of various combinations of three ion channel inhibitors for treatment of secondary degeneration: using lomerizine hydrochloride (Lom) [45], zonampanel monohydrate YM872 [46, 47] (also referred to as INQ) and/or oxidized ATP (oxATP) [48] to inhibit VGCCs, Ca^2+^ permeable AMPARs and P2X_7_Rs, respectively. We have demonstrated that the three ion channel inhibitors in combination were required to preserve myelin compaction, node of Ranvier length and behavioural function, in optic nerve vulnerable to secondary degeneration in the chronic phase, 3 months following injury [49]. However, in the acute phase after injury, it is not yet known whether ionic flux through particular channels is associated specifically with oxidative stress, axon degeneration, altered nodes and/or OPC loss. Here we used the partial optic nerve transection model and assessed early cytoskeletal protein and Nogo-A changes in the first week after injury. In vivo administration of various combinations of the three ion channel inhibitors was used as a tool to determine the relative importance of influx of extracellular ions through specific channels, for acute disruptions to visual behaviour, axon and node of Ranvier structure, oxidative stress and OPC numbers. Outcomes of inhibitor treatment were assessed at three days following injury as this is a time point known to feature oxidative damage, nodal disruptions and OPC responses in nerve vulnerable to secondary degeneration [30, 34], and were related to chronic rescue of myelin structure and function by the inhibitors in this model [49]. Outcome measures were confined to those known to change in the early phase of injury rather than those that appear chronically; an immunohistochemistry based approach was employed to enable assessment of cell numbers, structural components and cell-specific responses to injury.

## Methods

### Animals, anaesthesia and surgery

Female Piebald Virol Glaxo (PVG) adult rats (160–200 g), obtained from the Animal Resource Centre (Murdoch, Western Australia), were housed in groups of three under standard conditions including 12 h (h) light/dark cycles and ad libitum access to chow and water. All procedures were carried out in accordance with National Institutes of Health guide for the care and use of Laboratory animals (NIH Publications No. 8023, revised 1978) and approved by The University of Western Australia Animal Ethics Committee, Approval No. RA3/100/673. Female rats were used in order to complement our body of work on secondary degeneration following partial optic nerve transection in female PVG rats [6, 35, 49, 50]. Using a single sex of animals minimises variability due to sex specific differences and female animals can readily be group housed, making them convenient to work with. Anaesthesia was administered intraperitoneally (i.p.) as a combination of Xylazine (Ilium Xylazil 20, 10 mg/kg, Troy Laboratories) and Ketamine (Ketamil, 50 mg/kg, Troy Laboratories). Partial transection of the optic nerve, in which retinal ganglion cell (RGC) axons in the dorsal aspect of the optic nerve are lesioned leaving those on the ventral side intact but susceptible to secondary degeneration, was conducted as described previously [50]. Briefly, the dorsal side of the right optic nerve was partially transected approximately 1 mm behind the eye, to a controlled depth of 200 µm using a diamond radial keratotomy knife (Geuder). Post-operative analgesia was administered subcutaneously (2.8 mg/kg carprofen, Norbrook). Control animals were uninjured normal animals, as sham injured animals have been shown to be no different to normal in terms of visual function, RGC numbers and other cellular parameters [50]. Animals were euthanized with Euthal (active constituents Pentobarbitone Sodium 170 mg/mL, Phenytoin Sodium 25 mg/mL) at days 1, 3 or 7 post surgery.

### Treatments

Animals were randomly allocated into groups within three separate cohorts (described below) for testing of combinations of the three Ca^2+^ channel inhibitors, lomerizine (Lom; LKT Laboratories), OxATP (Sigma-Aldrich) and/or YM872 (LKT Laboratories). Treatment began on the day of surgery after full recovery from anaesthesia. Choices of treatment concentrations, routes and durations were based on previously published studies showing efficacy using these agents individually. Lom was administered orally twice daily in butter (30 mg/kg) [45]; all animals not receiving Lom received butter vehicle. OxATP (1 mM; Matute et al. [49]) and/or YM872 (240 µM) were dissolved in sterile phosphate buffered saline (PBS), and delivered at a rate of 0.5µL/h via a subcutaneously implanted, pre-loaded mini-osmotic pump (Model 2002; Alzet), attached to a cannula targeting the injured dorsal aspect of the optic nerve. Prior to the study, the stability of YM872 in the presence of PBS was assessed by reverse phase High Performance Liquid Chromatography (HPLC), on a Waters BEH C18 column with 275 nm UV detection. No degradation of YM872 dissolved in PBS was observed for 4 weeks at 37 °C. Similar analyses of oxATP showed some degradation after one day at 37 °C and almost complete degradation at 3 days, indicating that observed effects are due to the first day of oxATP treatment. Rats were housed individually to minimise disturbance of mini-pumps by cage-mates. Controls included injured animals treated with vehicle only (PBS in pumps and/or oral butter vehicle) and completely normal animals.

### Optokinetic nystagmus

At 2 days post-surgery, a cohort of rats (cohort 2: 8/group, n = 48 animals) were anaesthetised as above and their uninjured left eye lids sutured shut. Following complete recovery from anaesthesia (Day 3), behavioural testing was conducted using the optokinetic nystagmus test for visual function as described previously [50, 51]. Briefly, after acclimatisation, responses of rats to rotation of black and white stripes in the anti-clockwise direction were recorded. Analysis was performed by counting the number of purposeful movements in the direction of the stripes within the period each rat was engaged in the task.

### Tissue preparation

Tissue was collected from three separate cohorts of animals. Cohort 1 had 10 animals/group, total n = 30 animals, used for immunohistochemistry assessments of axonal changes at days 1 and 7 following injury, relative to normal control animals. Cohort 2 had 8 animals/group, total n = 48 animals, used for behavioural analyses and immunohistochemistry assessments of axonal and myelin structure following injury and treatment with ion channel inhibitor combinations or vehicle control, assessed at day 3. Cohort 3 had 5 animals/group, total n = 30, used for analyses of oxidative damage following injury and treatment with ion channel inhibitor combinations or vehicle control, assessed at day 3. For cohort 1 and 3, animals were euthanized with Euthal (Pentobarbitone sodium 850 mg/kg Phenytoin sodium 125 mg/kg; Delvet i.p.) and transcardially perfused with 0.9% NaCl followed by 4% paraformaldehyde (Sigma) in 0.1 M phosphate buffer, pH 7.2 (PFA). For cohort 2, animals were anaesthetised using ketamine–xylazil as described above, right optic nerves were dissected from the ocular cavity, collected into a drop of optimal cutter temperature compound (Tissue-Tek, Sakura), on a microscope slide, quickly frozen over a bed of dry ice, then snap-frozen in Eppendorf microcentrifuge tubes in liquid nitrogen in accordance with optimal conditions required for immunohistochemistry using specific node/paranode antibodies (Neurofascins: note that staining was equivocal and is not described further), and stored in airtight zip-lock bags at −80 °C, to avoid desiccation.

### Immunohistochemical assessments

Tissue was cryosectioned longitudinally at −20 °C (20 µm). Tissue from cohort 2 was fixed in 50:50 methanol/acetone for 15 min (min), washed with PBS, post-fixed for 1 min with Bouins solution and again washed with PBS prior to primary antibody application overnight at 4 °C. Immunohistochemical analyses were conducted according to established procedures [52], using primary antibodies recognising: manganese superoxide dismutase (MnSOD, 1:500; Abcam rabbit, SOD-110); DNA oxidation indicator 8-hydroxyguanosine (8OHDG, 1:500; Abcam mouse Ab62623); advanced glycation end-product carboxy-methyl lysine (CML, 1:500; CosmoBio, KAL-KH024); lipid peroxidation products 4-hydroxynonenal (HNE, 1:200; Alpha Diagnostics, rabbit HNE11-S), and Acrolein (1:1000; Abcam, rabbit Ab37110); protein nitration indicator 3-nitrotyrosine (3NT, 1:500; Abcam mouse Ab61392); myelin basic protein (MBP, 1:500; Abcam, rabbit Ab40390 or Santa Cruz, goat SC13914); β-III tubulin (1:500; Covance, mouse MMS-435P); ED1 for activated microglia/macrophages (CD68, 1:1000 Merk Millipore, mouse MAB1435); Iba1 for resident microglia/macrophages (1:1000 Abcam, goat Ab5076); Caspr (1:500, Abcam, rabbit Ab34151 or NeuroMab mouse 75-001); and Ankyrin G (AnkG, 1:200; Invitrogen, mouse 33-8800) for paranode and node of Ranvier structures; axonal components Tau (1:400, Invitrogen, mouse AHB0042), Tau p[S^396^] (1:200, Invitrogen rabbit 44752G), Tau p[S^262^] (1:400, Invitrogen, rabbit 44750G), Tau p[T^205^] (1:200, Invitrogen, rabbit 44738G), acetylated α-tubulin (1:500, Sapphire, mouse Ab24610), and NogoA (1:400, Millipore, rabbit Ab5888); olig2 (1:500, R and D Systems, goat AF2418) and NG2 (1:400, Merck Millipore, rabbit Ab5320 or mouse MAB5384) to identify OPCs. Antibody binding was visualized following 2 h incubation at room temperature with appropriate Alexa Flour 488 or 555 secondary antibodies (1:400; Molecular Probes, Life Technologies). Slides were cover-slipped using Fluoromount-G (Southern Biotechnology) and viewed using fluorescence microscopy.

### Immunohistochemistry image analysis and quantification

Assessment of immunointensity in optic nerve sections was semi-quantitative, using established procedures [6, 30], and in line with best practice [53], in order to assess cell-type specific changes with injury and ion channel inhibitor treatment. In brief: a single image of the area directly ventral to the primary injury site was visualized and photographed using either a Leitz Diaplan fluorescence microscope (Leica, Germany) where cellular colocalisation was not required e.g. for oxidative stress measures, or a Nikon Eclipse Ti inverted microscope (Nikon Corporation) with a 20× objective or a 40×/1.3 N.A. oil immersion objective. For each outcome measure, all images were collected in a single session, with constant exposure and microscope settings to ensure uniformity of imaging parameters and consistency between measures. When using the Nikon microscope, a series of optical images at 0.5 μm increments along the z-axis were acquired from the middle 6 μm of each 20 μm section, sampling a field of view of 217.5 × 162.5 μm (for 40× objective) of the ventral area, vulnerable to secondary degeneration. All images were collected using Nikon Elements AT software and deconvoluted using autoquant blind deconvolution. Deconvolution was performed using a custom written macro (Nathanael Yates) using the AQI_DeconvolutionND function and batch processing feature in NIS Elements. The images were then saved as new files for analysis; investigators were blinded to image identity. Image analysis was performed using a custom written macro in ImageJ. Briefly, each image was opened, and the visual slice along the z axis that was most in focus was selected for analysis. The image was then cropped to standard dimensions (500 × 500 pixel**)** in order to only include the region of interest. The intensity above an arbitrary set threshold, and area above that threshold were measured with constant parameters for all files. Choice of threshold was based on a visual determination of the level that captured clearly immunopositive areas in a selection of images from all groups. Note that the degree of changes to outcomes did not change with the deconvolution process. A range of normalisation techniques are employed in the literature to account for variation in tissue sections, and choice of these must be carefully considered in light of the outcome measure being assessed and the model and analyses employed. For immunohistochemical intensity and area data, such as in the current study, normalisation to background staining within the same section can be employed to account for variation in section thickness and antibody application [54]. Here, all immunointensity data assessing cellular structures was normalised to background. Note that normalisation to background is not possible for oxidative stress immunointensity data, as the oxidised proteins and DNA are diffusely distributed throughout the cells and/or tissue and there is nowhere in the tissue that can be conclusively described as background. Following injury, it is not appropriate to normalise data relative to other proteins as these can change with the pathological state. For example, β-III tubulin immunointensity increases at 3 days after injury [52]. As such, normalising to tubulin would result in inappropriate interpretation of data. The choice of data to display in histograms displaying immunointensity was based upon the pattern of immunoreactivity and whether changes occurred due to an increase in the area of immunofluorescence or an increase in the intensity of that immunofluorescence, as previously described [6]. For analyses of phosphorylated Tau (Tau p) [S^396^], Tau p[T^205^] or Tau p[S^262^], the same area was used to analyse immunointensities, and data were expressed as a ratio of total Tau immunointensity.

For node/paranode analyses, 30 node/paranode complexes were assessed from a single defined and consistent field of view from a single z-series of images from each animal, collected as described above, measuring: the length of the paranodal gap, defined as the distance between two Caspr^+^ paranodes [55]; the average length of the flanking paranodes (Caspr^+^); and AnkG length/distribution, defined as the length of AnkG^+^ staining between flanking paranodes. A representative orthogonal z-projection is shown to illustrate that the majority of node/paranode complexes did not span more than three visual slices; each z-stack was assessed to ensure inclusion of all elements of the node/paranode complex within the visual sampling before analysis. Axons of the optic nerve are predominantly medium caliber (mean ± SD = 0.64 ± 0.29) [56]; as such, the imaging strategy employed enabled detection of the majority of axons. NG^2+^/olig^2+^ OPCs were identified by colocalisation of immunoreactivity and the number of OPCs in a single visual slice of a single image of ventral optic nerve for each animal, collected as described above, was counted. A single visual slice was utilised to ensure co-localisation of NG2 and olig2. Numbers of ED1^+^ activated microglia/macrophages and Iba1^+^ resident microglia were counted from a single visual slice of single images from both dorsal and ventral optic nerve for each animal, collected as described above.

### Statistical analyses

Results were analysed using the statistical package Statview for Windows (SAS Institute Inc.) or IBM SPSS Statistics. Equality of variance F-tests were conducted to test for homogeneity of variance in groups within experiments. Data were natural log transformed, where necessary to achieve a normal distribution. All data are expressed as means of each treatment group ±SEM, unless otherwise stated. ANOVAs followed by Dunnett’s or Games Howell post hoc tests as appropriate were used to statistically compare quantitative measures of each treatment group to the injured vehicle control. For selected outcomes of interest, multiple comparisons using Bonferroni post hoc tests were performed and noted in the Results text. ANOVA F-test and degrees of freedom (df), as well as p value from post hoc tests are given. All statistical tests required p ≤ 0.05 for significance.

## Results

### Disruptions to axonal and oligodendrocyte proteins in secondary degeneration

**Fig. 1:**
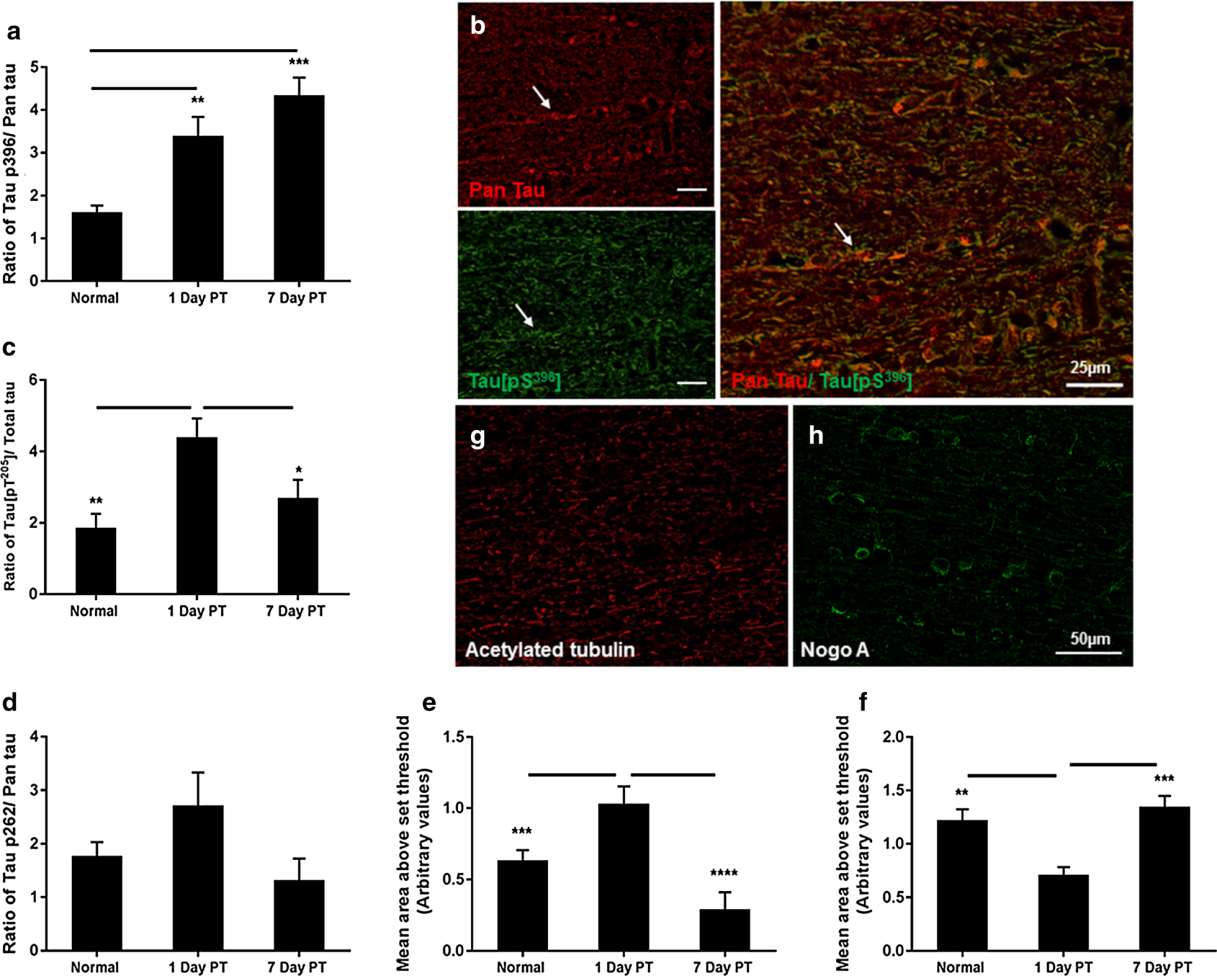
Indicators of cytoskeletal associated proteins assessed immunohistochemically in the ventral optic nerve at days 1 and 7 following partial transection (PT) injury. **a** The ratio of Tau p[S^396^] to total Tau immunoreactivity was calculated using mean ± SEM area above set threshold for each protein and quantified from normal optic nerve and at days 1 and 7 following partial transection; **b** representative images from normal optic nerve shows Tau p[S^396^] (*green*) and total Tau (*red*) with overlay, *arrows* indicate an example of co-localisation. **c** Similarly, the mean ± SEM ratio of Tau p[T^205^] to total Tau; and **d** the ratio of Tau p[S^262^] to total Tau ± SEM. **e** Mean ± SEM area above threshold of acetylated tubulin immunoreactivity; **f** mean ± SEM area above threshold of NogoA immunoreactivity. **g**, **h** Representative images from normal optic nerve show acetylated tubulin (*red*) and NogoA (*green*) respectively. Significant differences are indicated by *p < 0.05, **p < 0.01 and ***p < 0.001; **b** scale bar = 25 µm; **g**, **h** scale bar = 50 µm

Structural axonal and oligodendrocyte proteins were examined in ventral optic nerve vulnerable to secondary degeneration, following partial optic nerve transection. Note that the focus of the current study is on regions of the optic nerve vulnerable to secondary degeneration, rather than areas directly impacted by the primary injury. A significantly higher ratio of immunoreactivity of Tau p[S^396^] and Tau p[T^205^] relative to total Tau was observed in ventral optic nerve at day 1 following injury (F = 16.03, df = 2, p ≤ 0.01 and F = 7.08, df = 2, p ≤ 0.01 respectively), compared to normal optic nerve (Fig. 1a, c). While the ratio of Tau p[S^396^] to total Tau remained significantly higher at day 7 (Fig. 1a, b; p ≤ 0.001), the ratio of Tau p[T^205^] to total Tau was significantly reduced (Fig. 1c; p ≤ 0.001), returning to normal optic nerve levels. Changes in the ratio were driven by trends towards decreases in total Tau levels and increases in the levels of Tau phosphorylation, neither of which reached statistical significance (p > 0.05). There was no significant difference in the ratio of Tau p[S^262^] relative to total Tau in ventral optic nerve (Fig. 1d; F = 2.59, df = 2, p > 0.05), nor differences in Tau p[S^262^] immunoreactivity alone (p > 0.05). Immunoreactivity of acetylated tubulin was also significantly elevated in optic nerve vulnerable to secondary degeneration at day 1 following injury, returning to normal levels at day 7 (Fig. 1e, g; F = 16.49, df = 2, p ≤ 0.001). In contrast, NogoA immunoreactivity was significantly decreased at day 1 post injury in ventral optic nerve, and returned to normal levels at 7 days post injury (Fig. 1f, h; F = 13.79, df = 2, p ≤ 0.001).

### No acute effects of ion channel inhibitor combinations on behavioural deficits

**Fig. 2:**
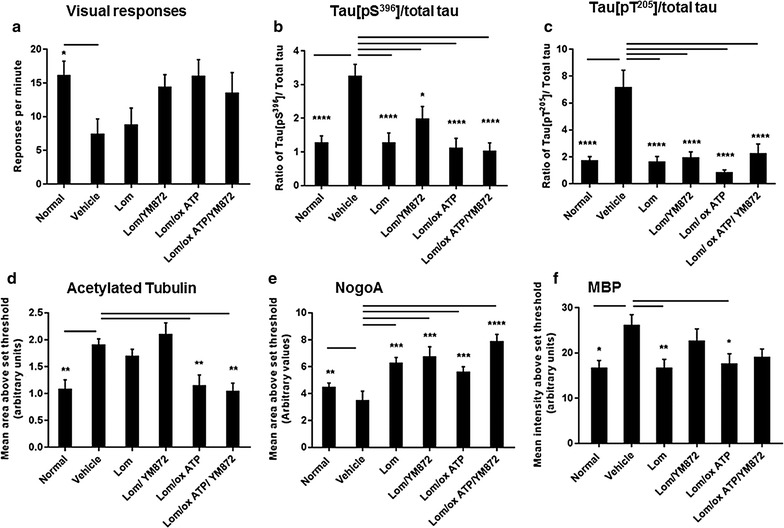
Mean ± SEM responses in the optokinetic nystagmus test of visual function and immunoreactivity of axonal and oligodendrocyte proteins, 3 days following partial transection of the optic nerve. **a** Total number of smooth pursuits and fast resets/minute engaged in the task by normal, or injured vehicle or inhibitor treated animals. **b** Effects of injury ± combinations of ion channel inhibitors on ratio of Tau p[S^396^] to total Tau and **c** ratio of Tau p[T^205^] to total Tau immunoreactivities were calculated using mean ± SEM area above an arbitrarily set threshold for each protein. Similarly, **d** mean ± SEM area above threshold of acetylated tubulin, **e** NogoA and **f** mean ± SEM intensity above threshold of MBP immunoreactivity. Significant differences compared to vehicle are indicated by *p < 0.05, **p < 0.01, ***p < 0.001 and ****p < 0.0001

Partial optic nerve transection resulted in a significant reduction in the number of optokinetic nystagmus responses at 3 days after injury (Fig. 2a; F = 2.54, df = 5, p ≤ 0.05). Despite a strong trend to increasing function with more inhibitors, treatment with a selection of combinations of ion channel inhibitors had no significant effect on behavioural responses at this acute phase following injury, when compared to vehicle treated animals (p > 0.05), in contrast to our reported preservation of visual function with the three inhibitors in combination at 3 months after injury [49]. Animals treated with more than one ion channel inhibitor made an intermediate number of responses, neither significantly improved above vehicle control nor different from normal animals (p > 0.05). Note that throughout the current study, outcomes of the different treatment combinations are not compared to each other. Furthermore, no detrimental effects of the inhibitor combination on animal welfare were observed.

### Effects of ion channel inhibitors on axonal and oligodendrocyte proteins

Similarly to findings at day 1 after injury, the ratios of Tau p[S^396^] and Tau p[T^205^] relative to total Tau in ventral optic nerve vulnerable to secondary degeneration were significantly increased at day 3 following injury, compared to normal optic nerve (Fig. 2b, c; F = 9.06, df = 5, p ≤ 0.001 and F = 12.39, df = 5, 33, p ≤ 0.001 respectively). All tested ion channel inhibitor combinations significantly reduced immunoreactivity of both Tau p[S^396^] and p[T^205^] expressed as a ratio of total Tau, when compared to vehicle treated animals (Fig. 2b, c; p ≤ 0.05); changes in pTau or total Tau alone were not significant (p > 0.05). Immunoreactivity of Tau p[S^262^] was not significantly altered at day 3 following injury, and there were no significant differences with ion channel inhibitors.

Similarly to findings at day 1 after injury, the immunoreactivity of acetylated tubulin was significantly elevated in vehicle treated animals 3 days post injury, compared to normal optic nerve (Fig. 2d; F = 8.80, df = 5, p ≤ 0.01). The combinations of Lom/oxATP and the three inhibitors significantly reduced acetylated tubulin immunoreactivity (p ≤ 0.01): Lom and Lom + YM872 resulted in maintenance of significantly elevated acetylated tubulin immunoreactivity above normal (p ≤ 0.01). There was a significant decrease in NogoA immunoreactivity in ventral optic nerve from vehicle treated animals, compared to normal optic nerve (Fig. 2e; F = 9.59, df = 5, p ≤ 0.05). Treatment of animals with all combinations of inhibitors significantly increased immunoreactivity of NogoA above vehicle treated animals (p ≤ 0.001). Interestingly, multiple comparison tests revealed that treatment with the triple combination of ion channel inhibitors resulted in significant upregulation of the expression of NogoA compared to normal nerve (Fig. 2e; p ≤ 0.02). There was a significant increase in MBP immunostaining intensity 3 days after injury in vehicle treated animals (Fig. 2f; F = 3.46, df = 5, p ≤ 0.05). Following treatment with selected ion channel inhibitor combinations, intermediate effects were observed, with only Lom, and Lom + oxATP treatments resulting in significant decreases compared to vehicle treated animals (Fig. 2f, p ≤ 0.05).

### Effects of ion channel inhibitor combinations on structure of the node of Ranvier complex

**Fig. 3:**
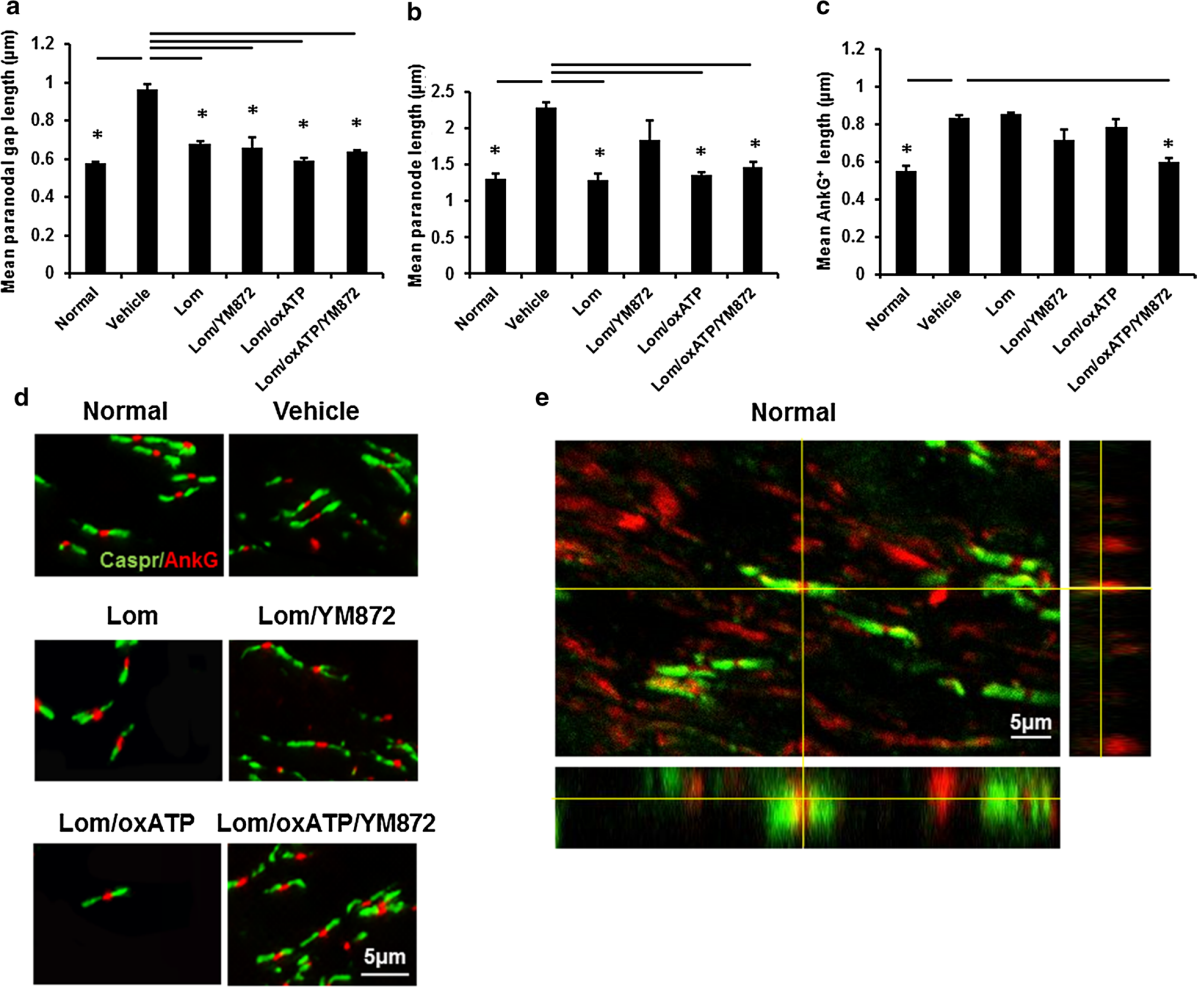
Effects of combinations of ion channel inhibitors on node/paranode complexes. Mean ± SEM **a** length of the paranodal gap, indicative of node length; **b** paranode length and **c** AnkG^+^ length were quantified from normal, or injured vehicle or inhibitor treated animals, 3 days following partial optic nerve transection. Significant differences relative to vehicle are indicated by *p ≤ 0.05. **d** Representative images show Caspr immunopositive paranodes (*green*) and AnkG immunopositive structures within the node of Ranvier (*red*); scale bar = 5 µm. **e** A representative orthogonal z-projection of Caspr immunopositive paranodes (*green*) and AnkG immunopositive structures within the node of Ranvier (*red*) from normal ventral optic nerve; scale bar = 5 µm

Structural parameters of the nodes of Ranvier and of paranodes were quantified in ventral optic nerve, and the effects of the selected combinations of ion channel inhibitors assessed. Note that analysis is confined to node/paranode structures able to be visualised using the imaging parameters employed, likely to include those associated with the majority of optic nerve axons [56]. Both the paranodal gap (F = 4.19, df = 5, p ≤ 0.05) and the average paranodal length (F = 3.12, df = 5, p ≤ 0.05) were significantly increased 3 days following injury compared to normal nerve (Fig. 3a, b, d). All of the tested ion channel inhibitor combinations resulted in significantly decreased paranodal gaps in ventral optic nerve, compared to vehicle treated animals (Fig. 3a, d; p ≤ 0.05). Similarly, most ion channel inhibitor combinations resulted in decreased paranode lengths (Fig. 3b, d; p ≤ 0.05); although outcomes following treatment with Lom + YM872 were not significantly different from vehicle treated animals or normal (Fig. 3b; p > 0.05). The average length of AnkG immunoreactivity at the node of Ranvier was significantly increased 3 days following injury, compared to normal (Fig. 3c, d; F = 15.237, df = 5, p ≤ 0.05), indicating a spread of Na^+^ channels in optic nerve vulnerable to secondary degeneration. Following treatment with selected ion channel inhibitor combinations, intermediate effects of Lom + YM872 and Lom + oxATP were observed, with only treatment with Lom + oxATP + YM872 resulting in significantly decreased lengths of AnkG immunoreactivity compared to vehicle treated animals (Fig. 3c, d; p ≤ 0.05). A representative orthogonal z-projection from normal optic nerve is shown (Fig. 3e): all node/paranode complexes assessed fell within the visual sampling.

### Effects of ion channel inhibitor combinations on microglia/macrophage numbers

The numbers of ED1^+^ activated microglia/macrophages and Iba1^+^ resident microglia/macrophages were quantified in both dorsal optic nerve directly impacted by the injury and ventral optic nerve vulnerable to secondary degeneration, as macrophage derived reactive species from dorsal nerve are thought to trigger secondary degeneration [6]. The numbers of ED1^+^ and Iba1^+^ cells were increased following injury, in both dorsal and ventral aspects of the optic nerve, as expected [52] (F = 13.902, F = 14.79 respectively, df = 5, p ≤ 0.05). However, there were no significant effects of any of the inhibitor combinations on numbers of ED1^+^ or Iba1^+^ microglia/macrophage (Table 1, p > 0.05).

**Table 1:** Numbers of ED1^+^ and Iba1^+^ microglia/macrophages in dorsal and ventral optic nerve following partial transection

	Normal	Vehicle	Lom	Lom/YM872	Lom/oxATP	Lom/oxATP/YM872
*ED1*						
Dorsal	5.4 ± 0.8	34.5 ± 11.2*	33.5 ± 10.5	29.0 ± 5.8	47.0 ± 11.3	38.6 ± 11.0
Ventral	5.0 ± 1.1	22.5 ± 5.5*	16.8 ± 4.9	26.1 ± 8.6	32.0 ± 9.5	33.2 ± 9.7
*Iba1*						
Dorsal	5.2 ± 1.0	19.2 ± 4.5*	21.1 ± 4.9	29.6 ± 3.6	32.8 ± 4.7	29.2 ± 3.9
Ventral	4.8 ± 1.0	14.1 ± 1.7*	9.5 ± 2.7	27.4 ± 6.8	24.8 ± 4.7	28.9 ± 4.4

Mean ± SEM numbers of ED1^+^ and Iba1^+^ cells within the field of view; significant differences relative to vehicle are indicated by * p ≤ 0.05

### Effects of ion channel inhibitor combinations on oxidative stress indicators and OPCs

**Fig. 4:**
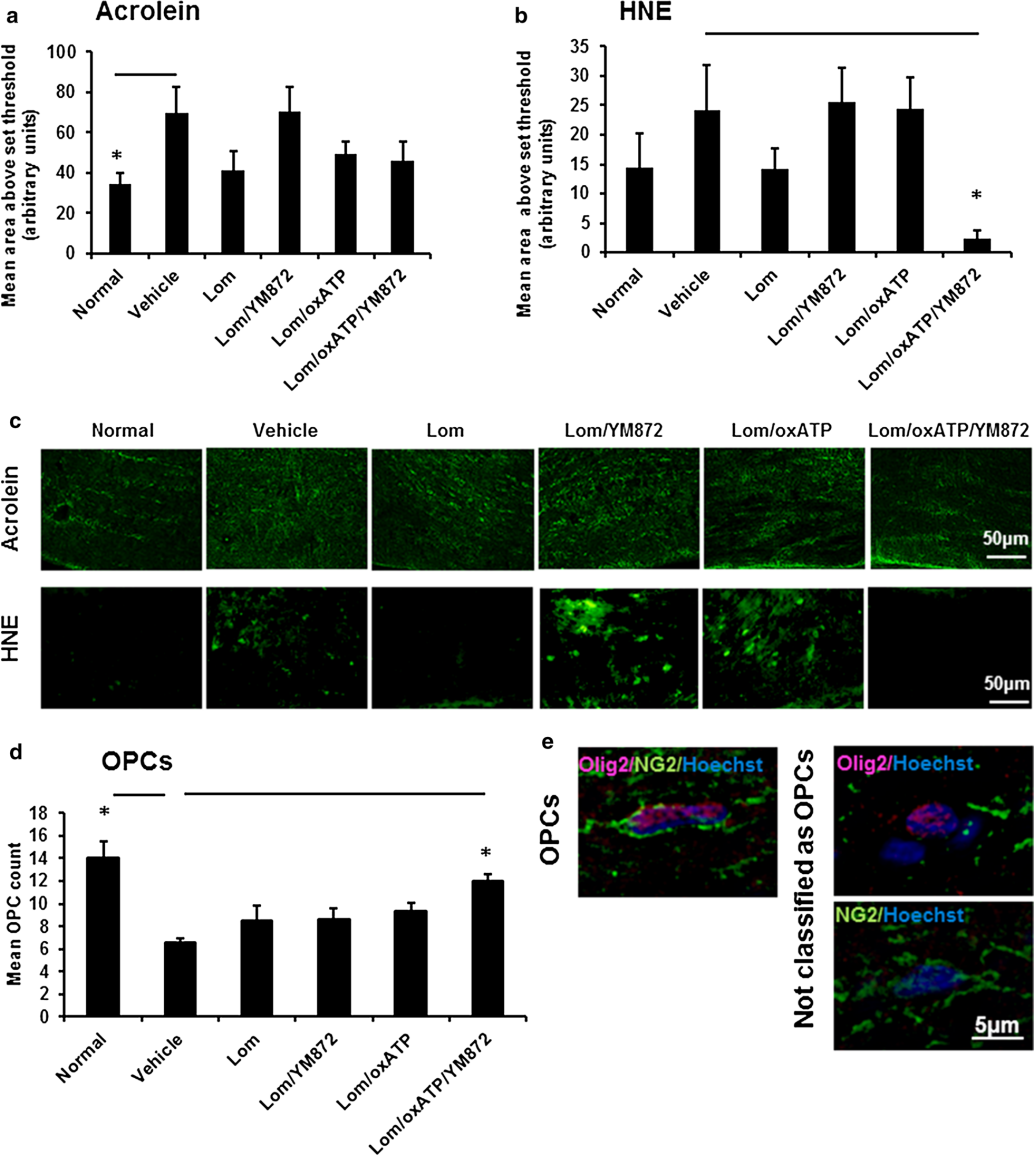
Effects of combinations of ion^+^ channel inhibitors on oxidative stress and oligodendrocyte progenitor cells (OPCs). Mean ± SEM area above an arbitrarily set threshold **a** acrolein and **b** HNE immunointensity above an arbitrarily set threshold, in ventral optic nerve from normal, or injured vehicle or ion inhibitor treated animals, 3 days following partial optic nerve transection. Significant differences relative to vehicle are indicated by *p ≤ 0.05. **c** Representative images of acrolein and HNE immunointensity are shown; scale bar = 50 µm. **d** Mean ± SEM oligodendrocyte progenitor cell (OPC) counts in ventral optic nerve from normal, or injured vehicle or inhibitor treated animals, 3 days following partial transection. **e** Representative images illustrating OPC identification as NG2^+^/olig2^+^ cells; scale bar = 5 µm

An increase in the immunopositive areas of lipid peroxidation product acrolein (Fig. 4a, c; F = 2.57, df = 5, p ≤ 0.05) was observed in optic nerve of vehicle treated animals 3 days following partial transection. This was associated with a significant decrease in the number of Olig2^+^/NG2^+^ OPCs (Fig. 4d; F = 4.03, df = 5, p ≤ 0.05). There was no significant effect of injury on immunointensity of the lipid peroxidation indicator HNE at three days following injury (Fig. 4b, c; F = 2.86, df = 5, p > 0.05). However, HNE was significantly reduced following treatment with Lom + OxATP + YM872 (p ≤ 0.05): other inhibitor combinations were not significantly different from vehicle treated animals or normal (p > 0.05). Similarly, only treatment with Lom + OxATP + YM872 significant increased OPC numbers relative to vehicle control (Fig. 4d; p ≤ 0.05; Fig. 4e shows representative images of OPCs). There was an intermediate effect of all combinations of ion channel inhibitors on acrolein, with no statistically significant difference between normal or injured vehicle treated nerve with any ion channel inhibitor treatment group (Fig. 4a, p > 0.05).

**Fig. 5:**
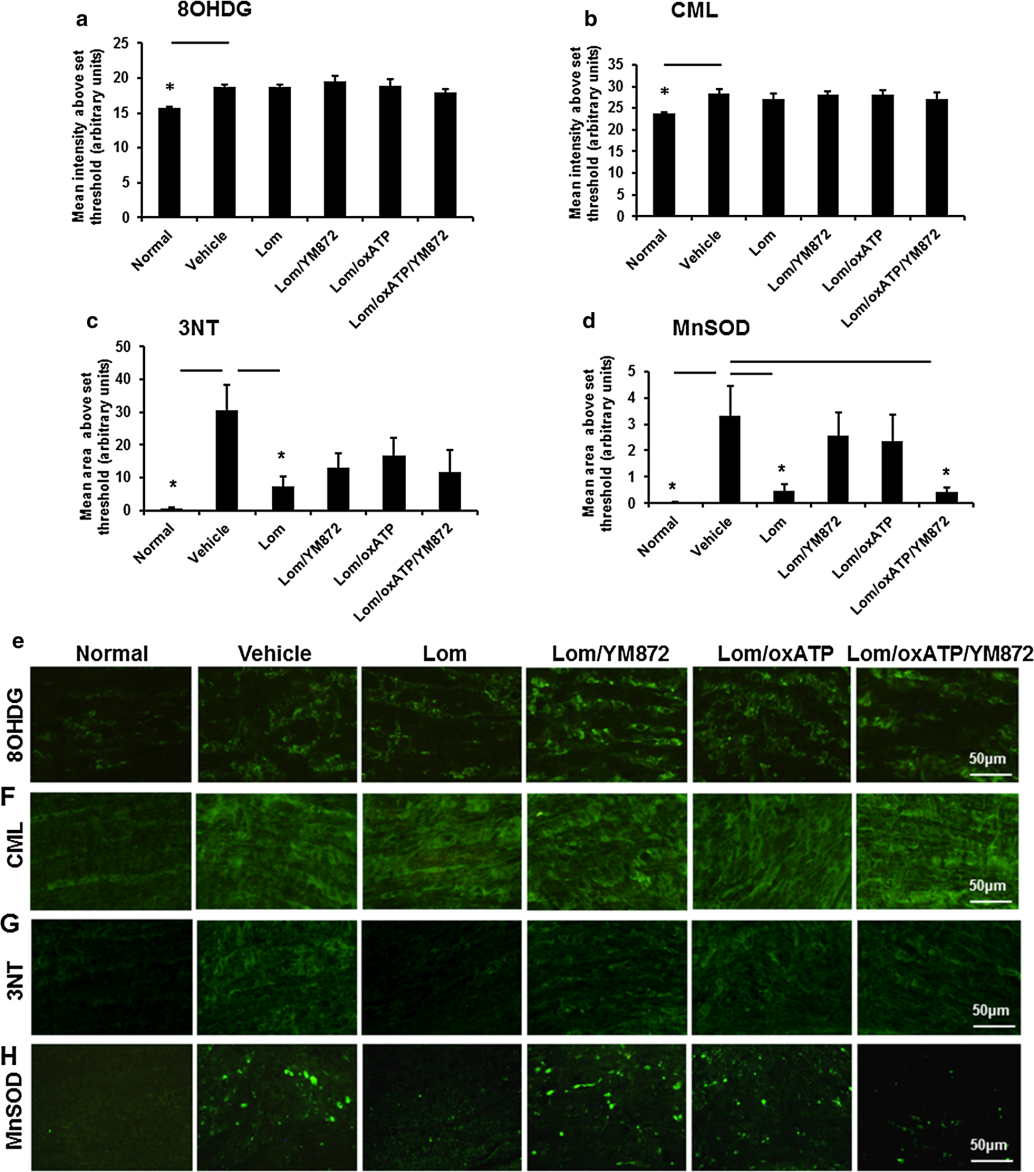
Effects of combinations of ion channel inhibitors on additional indicators of oxidative stress. Mean ± SEM intensity above an arbitrarily set threshold for **a** 8-hydroxy-2′deoxyguanosine (8OHDG); **b** Carboxymethyl lysine (CML); and mean ± SEM area above an arbitrarily set threshold for **c** 3-nitrotyrosine (3NT); or **d** manganese superoxide dismutase (MnSOD) immunoreactivity were quantified at 3 days following partial optic nerve transection, in ventral optic nerve of normal, or injured vehicle or treated animals. Significant differences relative to vehicle are indicated by *p ≤ 0.05. Representative images of **e** 8OHDG, **f** CML, **g** 3NT and **h** MnSOD immunoreactivity in normal and injured vehicle or inhibitor treated ventral nerve, 3 days following partial transection; scale bars = 50 μm

Significant increases in the mean intensity of immunoreactivity of DNA oxidation marker 8OHDG (Fig. 5a, e; F = 3.82, df = 5, p ≤ 0.05), advanced glycation end-product CML (Fig. 5b, f; F = 2.27, df = 5, p ≤ 0.05), protein nitration indicator 3NT (Fig. 5c, g; F = 5.23, df = 5, p ≤ 0.05) and antioxidant enzyme MnSOD (Fig. 5d, h; F = 2.33; df = 5, p ≤ 0.05) were observed in optic nerve vulnerable to secondary degeneration, 3 days following partial transection. MnSOD immunoreactivity displayed a punctate pattern, likely reflecting aggregates of cellular debris as previously described [50]. Following treatment with the selected ion channel inhibitor combinations, no significant reductions in 8OHDG or CML were observed, relative to vehicle treated animals (Fig. 5a, b, p > 0.05). Only Lom treatment significantly reduced 3NT immunoreactivity, whereas both Lom and Lom + OxATP + YM872 treatment reduced MnSOD immunoreactivity (Fig. 5c, d; p ≤ 0.05). For 8OHDG, all inhibitor combinations other that Lom + YM872 + oxATP remained significantly increased from normal (p ≤ 0.05); other inhibitor combinations resulted in an intermediate effect for CML, 3NT and MnSOD, not significantly different from normal or vehicle (p > 0.05).

## Discussion

Changes in Ca^2+^ dynamics during secondary degeneration have been shown to be associated with oxidative stress and disruptions to myelin structure. Here we demonstrate acute increases in Tau protein phosphorylation, tubulin acetylation and decreases in NogoA in optic nerve exclusively vulnerable to secondary degeneration. Combinations of ion channel inhibitors were used as a tool to dissect out which ion channels are important for influx of extracellular ions associated with the disruptions of secondary degeneration. It was found that inhibition of most ion channel combinations assessed, including VGCCs alone, led to restoration of the normal lengths of the paranodal gap, and paranode and normal levels of phosphorylated Tau and NogoA. However, only inhibition of VGCCs, P2X_7_Rs and AMPARs together, significantly restored AnkG length at the nodes of Ranvier to normal, limited HNE immunoreactivity and prevented OPC loss (see summary of outcomes in Table 2). Inhibition of all three ion channels also leads to long term preservation of visual function in this model [49], implying an important role for these latter components of the injury response in chronic deficits.

**Table 2:** Summary of outcomes following partial transection injury and treatment with combinations of ion channel inhibitors to inhibit VGCCs, Ca^2+^ permeable AMPARs and/or P2X_7_Rs at 3 days after injury

Ion channels inhibited	Vehicle	VGCC	VGCC + Ca^2+^ permeable AMPAR	VGCC + P2X_7_R	VGCC + P2X7R + Ca^2+^ permeable AMPAR
Outcome measure					
Visual function	↓	~	~	~	~
Tau[pS^396^]/total Tau	↑	↓	↓	↓	↓
Tau[pT^205^]/total Tau	↑	↓	↓	↓	↓
Acetylated tubulin	↑	–	–	↓	↓
NogoA	↓	↑	↑	↑	↑
MBP	↑	↓	~	↓	~
Paranodal gap length	↑	↓	↓	↓	↓
Paranode length	↑	↓	~	↓	↓
Ankyrin G length	↑	–	~	~	↓
Microglia/macrophages	↑	~	~	~	~
Acrolein	↑	~	~	~	~
HNE	~	~	~	~	↓
OPCs	↓	~	~	~	↑
8OHDG	↑	↑	↑	↑	~
CML	↑	~	~	~	~
3NT	↑	↓	~	~	~
MnSOD	↑	↓	~	~	↓

Symbols for vehicle treated animals indicate direction of change from normal untreated animals. Following treatment with ion channel inhibitors, significant decreases relative to injured animals treated with vehicle are shown as ↓, significant increases as ↑, remaining significant differences to normal are indicated by—(p ≤ 0.05) and intermediate outcomes not significantly different from vehicle treated or normal as ~(p > 0.05)

Increased Tau phosphorylation reduces the binding of Tau to microtubules in intact neurons [57] and phosphorylation of Tau at [S^396^] reduces its affinity for and ability to stabilize microtubules [58]. While it is important to note that the number of specific phosphorylation sites recognised with the phosphorylation dependent antibodies we have used is limited, with over 25 phosphorylation sites in Tau that are associated with disease or injury [59] not assessed in the current study, some relationships can be explored. We observed no changes in the ratio of phosphorylated Tau at [S^262^] in tissue vulnerable to secondary degeneration, but other isoforms exhibited increased phosphorylated Tau. Tau phosphorylation specifically at [S^262^] has been reported to be independent of Ca^2+^ whereas phosphorylation of other Tau proteins is Ca^2+^ dependent [60]. Taken together, this suggests that the observed changes in Tau phosphorylation associated with secondary degeneration are induced by Ca^2+^ influx. The importance of Ca^2+^ influx in Tau phosphorylation in secondary degeneration is further supported by our findings of restored ratios of phosphorylated Tau to total Tau at [S^396^] and [T^205^] following treatment with all tested combinations of ion channel inhibitors. However, limiting flux of other ions such as Na^+^ through P2X_7_R and Ca^2+^ permeable AMPAR may also have contributed to observed effects. We have recently demonstrated that of the tested combinations of ion channel inhibitors, the Lom + YM872 and Lom + OxATP + YM872 combinations significantly reduced intracellular Ca^2+^ concentrations in mixed cortical cultures exposed to H_2_O_2_ insult [61]. While the in vitro environment is very different to the injured nerve in vivo and results are not directly comparable, it is likely that limiting excess Ca^2+^ flux is contributing to beneficial outcomes for phosphorylated Tau and other measures. Acetylated tubulin increases have been linked to calcium changes and may reflect increased axonal stability [62] in optic nerve vulnerable to secondary degeneration, although this is not supported by observed increases in Tau phosphorylation. The restoration of normal tubulin acetylation only by two or three ion channel inhibitors together, likely reflects restored ionic homeostasis. Nogo-A inhibits axonal extension and has been shown to decrease early after spinal cord injury, followed by later increases [38]. Similarly, we observed an acute decrease in NogoA immunoreactivity, which was prevented by all of the ion channel inhibitor combinations, indicating a generalised role for maintenance of intracellular ionic homeostasis in NogoA responses. Taken together, the data show that specific elements of axonal stability are regulated to differing degrees by altered ionic homeostasis.

An increase in the length of the node of Ranvier has been observed in many pathologies including multiple sclerosis [55], glutamate excitotoxicity [63], hyperoxia [64]; and spinal cord injury [65, 66]. It has been suggested that this lengthening may be due to myelin retraction and a breakdown of the paranodal junction [63, 67]. The node lengthens, whilst maintaining normal paranodal and juxtaparanodal structure, due to insertion of more membrane at the node [68]. Along the myelinated axon, an appreciable amount of Ca^2+^ influx is due to VGCCs, normally located and controlled at the axolemma [69, 70]. When the axolemma in these regions are exposed due to myelin retraction and splitting at paranodal regions, excessive influx of Ca^2+^ through axonal L-type VGCCs may occur [71]. Lomerizine inhibition of VGCCs, known to be present on myelinating mature oligodendrocytes [72], is associated with restoration of paranode structure. Inhibition of VGCCs, together with shielding of these channels by myelin, likely contributes to reduced Ca^2+^ mediated activation of Tau phosphorylation [60].

A generalised increase in MBP immunoreactivity was observed in optic nerve vulnerable to secondary degeneration, as previously described [52]. Increased MBP immunoreactivity has been shown to reflect a release of MBP into the surrounding milieu, followed by upregulated expression of MBP mRNA and protein [73], and/or conformational changes [74]. It is not yet clear why only Lom, and Lom + oxATP treatments resulted in reduced MBP immunoreactivity whereas other combinations of inhibitors did not. Disruption of myelin can cause increases in NG2 immunopositive cells [75]; however we have observed decreases in NG^2+^/olig^2+^ OPCs acutely in the current study, and chronically [34]. OPC depletion can be due to the cytotoxic action of the lipid peroxidation product 4-HNE on OPCs [76], supported by the observation that reductions in HNE via treatment with the three inhibitors in combination decreased both HNE immunoreactivity and the loss of OPCs in nerve vulnerable to secondary degeneration. HNE is both a product of lipid peroxidation and a toxic metabolite, observed to increase in optic nerve vulnerable to secondary degeneration [6]. While HNE was not significantly increased compared to normal in the current study, the reduction with treatment with the three inhibitors indicates a shift in the balance of oxidative metabolism below normal homeostasis and may reflect a compensatory response. 4-HNE treatment increases intracellular Ca^2+^ levels [77] and 4-HNE is toxic to axons and oligodendrocytes [78], covalently binding cytoskeletal proteins [79, 80], disrupting cytoskeletal structure [81], conjugating proteins [82, 83], and inhibiting mitochondrial respiration [83]. The associative relationship between the protection of OPCs and reduction in HNE immunoreactivity as a consequence of inhibiting VGCCs, P2X_7_Rs and Ca^2+^ permeable AMPARs, supports the importance of influx of extracellular Ca^2+^ and associated lipid peroxidation in OPC vulnerability. OPCs are reported to be particularly vulnerable to oxidative damage, with maturation dependent vulnerability [33] and a lack of intrinsic antioxidants [84]. Indeed, we do not observe either acute or chronic loss of mature CC1^+^ oligodendrocytes in this model [34]. Acrolein elimination requires GSH, inherently low in OPCs [84], which may explain why the lipid peroxidation indicator acrolein was not similarly reduced. Intermediate effects of ion channel inhibition were observed with a range of other elements of oxidative damage, indicating complex contributions of ions including Ca^2+^ to generation of reactive species and subsequent secondary degeneration.

The preservation of OPC numbers observed in the current study may have been due to increased proliferation above the baseline proliferative response to the injury that we have already reported [34], or reduced OPC death, and further studies will be required to investigate this mechanism. OPCs are thought to contact axons at the node of Ranvier and contribute to Na channel clustering [85]. In the current study, a continued spread of sodium channels, indicated by AnkG immunoreactivity [86] was observed, even when paranode structure was preserved by the less effective combinations of ion channel inhibitors. Our working hypothesis is that the protection of OPCs may contribute to AnkG clustering as a specific consequence of limiting HNE with the multiple ion channel inhibitors, thereby contributing to observed long term preservation of function [49]. The lack of significant preservation of acute function following treatment with various combinations of ion channel inhibitors, is associated with increased microglia/macrophages and alterations in MBP and selected oxidative stress indicators, some of which resolve as time passes [49]. Ca^2+^ influx modulated by Na ions and Na influx independent of Ca^2+^ [69, 87] may also contribute to acute pathology. In addition, potassium channels such as Kv2.1 can regulate Ca^2+^ influx [88] and have been shown to be oxidised following traumatic brain injury [89], which may further influence ionic flux. Internal Ca^2+^ stores released via ryanodine and inositol triphosphate receptors may also contribute to secondary degeneration [90]; ryanodine receptors are also oxidised by reactive species, thus leaking Ca^2+^, and likely further contributing to pathology [91]. Visual responses are affected by secondary degeneration of the ventral optic nerve. Protection of ventral degeneration can preserve visual function at normal levels, as assessed by both the optokinetic nystagmus and Y-maze pattern recognition task [49, 92], and despite axotomy of dorsal axons. It is likely that the disparity between acute visual deficits and chronic visual function rescue reflects transient disruptions to axonal transport and function that were later rescued with the combination of three inhibitors.

## Conclusions

Using ion channel inhibitors as a tool has allowed increased understanding of the multiple components of pathology that contribute to the acute phase of secondary degeneration.
